# Description of the supergiant isopod *Bathynomus raksasa* sp. nov. (Crustacea, Isopoda, Cirolanidae) from southern Java, the first record of the genus from Indonesia

**DOI:** 10.3897/zookeys.947.53906

**Published:** 2020-07-08

**Authors:** Conni M. Sidabalok, Helen P.-S. Wong, Peter K. L. Ng

**Affiliations:** 1 Division of Zoology, Research Center for Biology, Indonesian Institute of Sciences (LIPI), Gedung Widyasatwaloka, Cibinong Science Center, Jl Raya Jakarta-Bogor Km 46, Cibinong 16911, Indonesia Division of Zoology, Research Center for Biology, Indonesian Institute of Sciences (LIPI), Cibinong Science Center Bogor Indonesia; 2 St. John’s Island National Marine Laboratory, Tropical Marine Science Institute, National University of Singapore (NUS), 18 Kent Ridge Road, 119227, Singapore St. John’s Island National Marine Laboratory, Tropical Marine Science Institute, National University of Singapore (NUS) Singapore Singapore; 3 Lee Kong Chian Natural History Museum (LKCNHM), 2 Conservatory Drive, National University of Singapore, Singapore 117377, Singapore Lee Kong Chian Natural History Museum, National University of Singapore Singapore Singapore

**Keywords:** *
Bathynomus
*, Cirolanidae, Indian Ocean, Indonesia, new species, South Java, taxonomy

## Abstract

The giant isopod genus *Bathynomus* A. Milne-Edwards, 1879, is recorded for the first time in Indonesian waters, from deep waters off southern Java in the Indian Ocean. *Bathynomus raksasa***sp. nov.** is described and notes on juvenile specimens of an unidentified species found in the same locality are also provided. *Bathynomus raksasa***sp. nov.** is characterized by the large size (averaging at 330 mm), narrowly rounded clypeus apex, prominent longitudinal carina on the clypeus, convex lateral margins of the uropodal exopod and endopod, produced distolateral corners of the uropodal exopod and endopod which have acute ends, an uropodal exopod with a setal fringe of medium length (69%), a pleotelson 1.6 times wider than long with the posterior margin medially concave, and the large number (11–13) of spines on the pleotelson.

## Introduction

The genus *Bathynomus* A. Milne-Edwards, 1879 inhabits the deep sea in the Atlantic, Pacific and Indian Oceans, with some species reaching large sizes in excess of 30 cm length (Lowry and Dempsey 2006). Nineteen extant species are known in the genus (Bruce 1986, Magalhães and Young 2003, Lowry and Dempsey 2006, Boyko et al. 2008, Shipley et al. 2016, Kou et al. 2017).

Lowry and Dempsey (2006) revised the Indo-West Pacific taxa and recognized 16 species, of which seven were categorized as “supergiants”; species maturing above 150 mm and reaching 500 mm in length. Five “supergiant” species occur in the Indian and Pacific Oceans: *Bathynomus lowryi* Bruce & Bussarawit, 2004 (Andaman Sea), *B. crosnieri* Lowry & Dempsey, 2006 (Madagascar), *B. keablei* Lowry & Dempsey, 2006 (India, Sri Lanka, Burma), *B. kensleyi* Lowry & Dempsey, 2006 (Coral Sea, Philippines, South China Sea), and *B. richeri* Lowry & Dempsey, 2006 (New Caledonia) (Lowry and Dempsey 2006). Two other “supergiant” species are known from the western Atlantic: *B. giganteus* A. Milne-Edwards, 1879, and *B. miyarei* Lemos de Castro, 1978 (Boyko et al. 2008). The new species described here adds another “supergiant” *Bathynomus* from the Indian Ocean to this list, and is the first from Indonesia.

## Material and methods

**Figure 1.**
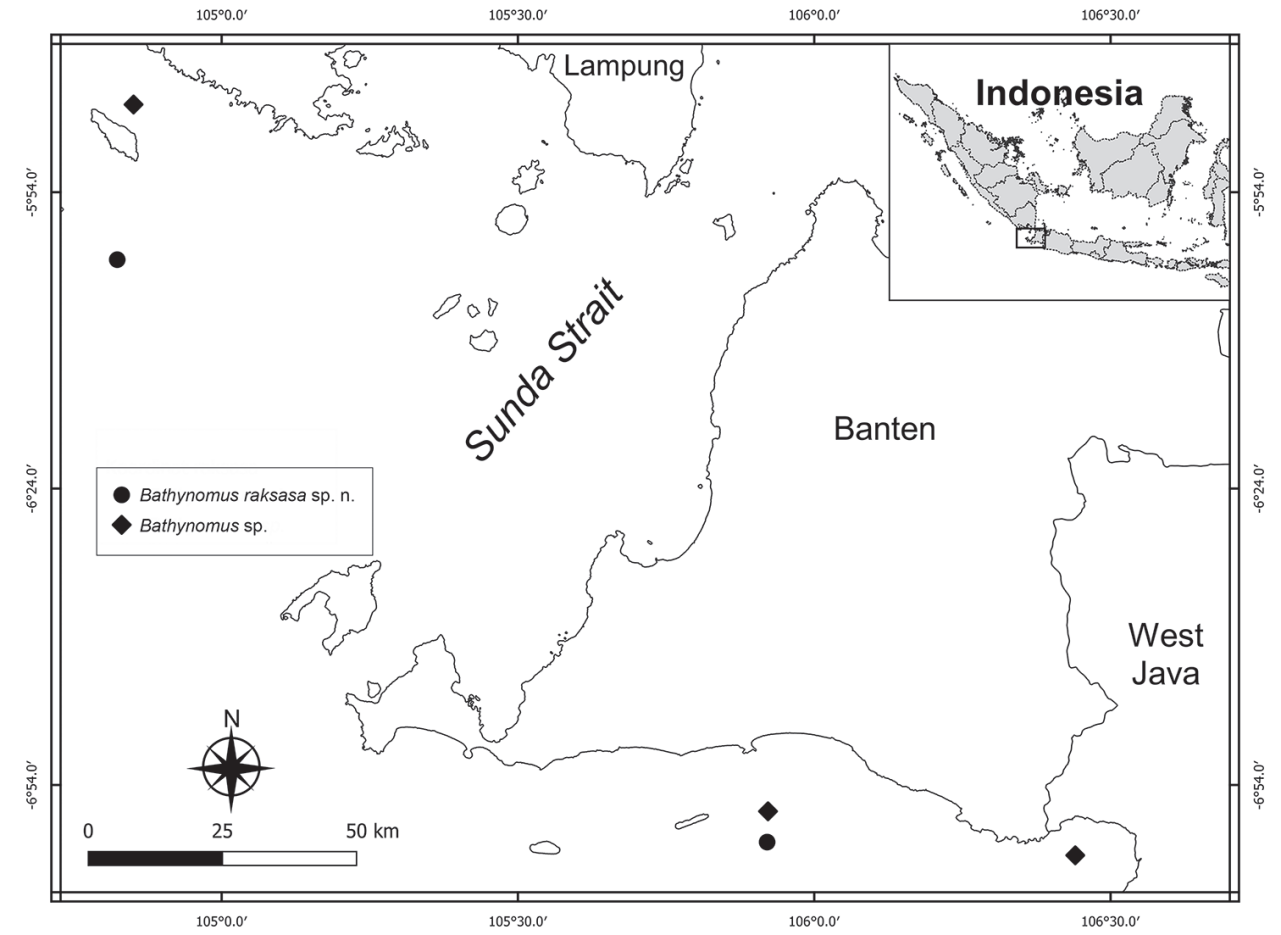
Distribution of *Bathynomus raksasa* sp. nov. and *Bathynomus* sp. in Indonesian waters.

The material was collected by the 2018 South Java Deep Sea Survey (SJADES 2018), a joint project between NUS and LIPI, with localities mostly in southern Sumatra and Java (Fig. 1). The terminology used and description format follows Lowry and Dempsey (2006).

The following acronyms are used: AM – Australian Museum, Sydney; LIPI – Lembaga Ilmu Pengetahuan Indonesia (Indonesian Institute of Sciences); MZB – Museum Zoologicum Bogoriense, Indonesia; NUS – National University of Singapore; SJADES – South Java Deep Sea Expedition; ZRC – Zoological Reference Collection of the Lee Kong Chian Natural History Museum, National University of Singapore.

## Taxonomy

**Suborder Cymothoida Wägele, 1989**

**Family Cirolanidae Da na, 1852**

### Genus Bathynomus

Taxon classificationAnimaliaIsopodaCirolanidae

A. Milne-Edwards, 1879

0068392D-5D33-593E-BC48-5EB50DFD27AE

#### Restricted synonymy.

A. Milne-Edwards, 1879: 21.— Bruce 1986: 126.— Kensley and Schotte 1989: 129.— Lowry and Dempsey 2006: 168.

#### Remarks.

The taxonomy of *Bathynomus* has been reviewed by Bruce (1986), Magalhães and Young (2003), with most recently by Lowry and Dempsey (2006). Two new species were added by Shipley et al. (2016) and Kou et al. (2017). The most recent review on *Bathynomus* fossils was done by Hyžný et al. (2019).

#### Type species.

*Bathynomus giganteus* A. Milne-Edwards 1879; by monotypy.

### Bathynomus raksasa sp. nov.

Taxon classificationAnimaliaIsopodaCirolanidae

2C1A752E-BD93-579E-8A09-338DED303E6C

http://zoobank.org/84D71359-90FB-4CC6-856F-B96402F23211

Figs 2 , 3 , 4 , 5

#### Material examined.

***Holotype***, male, 363 mm; Indonesia, Sunda Strait (between Sumatra and Java); 6°00.828'S, 104°49.428'E; 26 Mar. 2018; SJADES exped.; station CP 13, beam trawl 1259 m; MZB Cru.Iso 097. ***Paratype***, female, 298 mm; Indonesia, Indian Ocean (East of Tinjil Island); 6°59.778'S, 105°55.224'E; 28 Mar. 2018; SJADES exped.; station CP 28, beam trawl 957 m; ZRC 2020.0015.

#### Comparative material.

*Bathynomus giganteus* A. Milne-Edwards, 1879 – 1 male, 354 mm; U.S.A., Virginia, 100 miles off Virginia Beach; 36.483N, 74.8W; 30 May 1962; 73 m depth; ZRC 2014.0837. *Bathynomus doederleini* Ortmann, 1894 – 6 males, 100, 120, 120, 128, 136, 145 mm; 7 females, 88, 90, 94, 130, 130, 138, 145 mm; 3 juveniles; Taiwan; AM P68684. 1 male, 125 mm; 1 female, 85 mm; 4 juveniles; Taiwan, Tashi port; 1990s; P. K. L. Ng leg.; deep-water; ZRC 1998.417. *Bathynomus* sp. – 1 subadult, not sexually mature, pereopod 7 not fully developed, 107 mm; Indonesia, Indian Ocean (East of Tinjil Island); 6°56.664'S, 105°55.315'E; 28 Mar. 2018; SJADES exped.; station CP 26, beam trawl 517 m; MZB Cru.Iso 098. 1 juvenile; Indonesia, Sunda Strait (between Tabuan Island and Sumatra); 5°45.126'S, 104°51.080'E; 25 Mar. 2018; SJADES exped.; station CP 08, beam trawl 442 m; ZRC 2020.0016. 2 juveniles, 60, 63 mm; Indonesia, Indian Ocean (Pelabuhan Ratu Bay); 7°01.116'S, 106°26.421'E; 3 Apr. 2018; SJADES exped.; station CP 55, beam trawl 379 m; ZRC 2020.0017.

#### Type-locality.

Indonesia, Sunda Strait: between Sumatra and Java, 06°00.828'S, 104°49.428'E.

#### Diagnosis.

**Figure 2.**
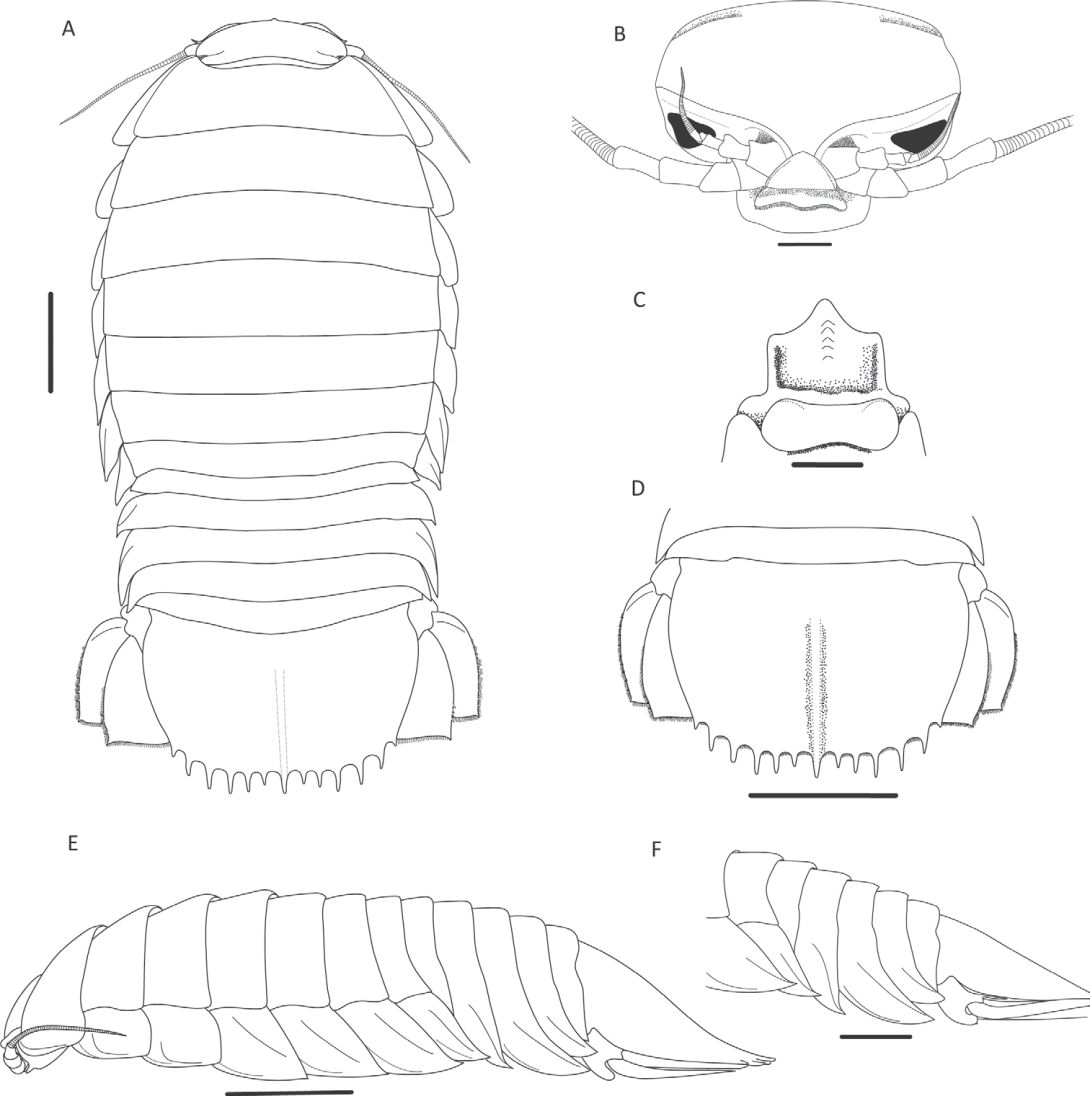
*Bathynomus raksasa* sp. nov., holotype male (363 mm) (MZB Cru.Iso 097), Indonesia **A** dorsal view **B** cephalon, anterior view **C** clypeal region **D** pleotelson **E** body, lateral view **F** pereon, lateral view. Scale bars: 5 cm (**A, D, E**);1 cm (**B, C, F**).

**Figure 3.**
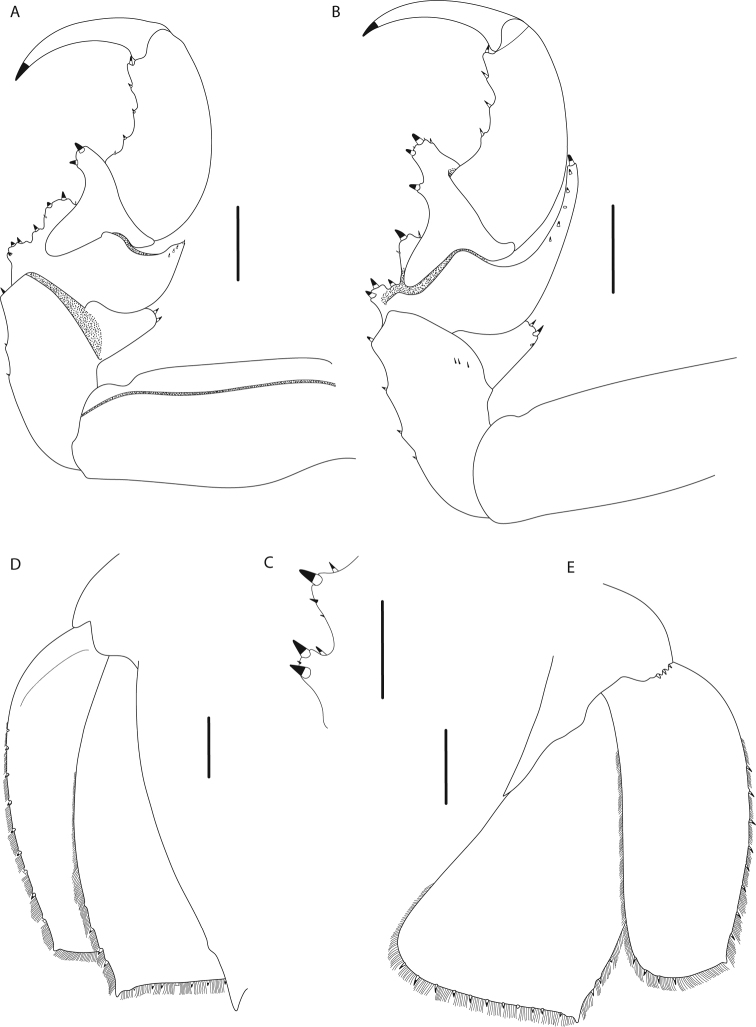
*Bathynomus raksasa* sp. nov., holotype male (363 mm) (MZB Cru.Iso 097), Indonesia **A** pereopod 1 **B** pereopod 2 **C** pereopod 2 merus, posterolateral margin **D** uropod, ventral view **E** uropod, dorsal view. Scale bars: 1 cm (**A, B, D, E**); 0.5 cm (**C**).

Narrowly rounded clypeus apex (Fig. 2C); prominent longitudinal carina on clypeus (Fig. 2C); convex lateral margins of uropodal exopod and endopod (Fig. 3D, E); produced distolateral corners of uropodal exopod and endopod with acute tips (Fig. 3D, E); uropodal exopod with medium-length setal fringe (69%) (Fig. 3D, E); pleotelson 1.6 times wider than long with posterior margin medially concave (Fig. 2D); 11–13 spines on pleotelson (Fig. 2D).

#### Description of holotype male.

Body (Fig. 2A) 363 mm long, 155 mm wide at pereonite 5, length 2.3 times width. Head (Fig. 2B) with ridge above eyes discontinuous; clypeus (Fig. 2C) with prominent longitudinal carina, distal margins slightly concave, apex narrowly rounded.

Antenna 2 (Fig. 2A, E) flagellum extending to end of pleonite 2.

Pereopod 1 (Fig. 3A) ischium with 2 posteroproximal robust setae, 2 robust setae on posterodistal margin; merus with 4 short robust setae on anterodistal angle, posterior margin with 4 robust setae in proximal row and 2 robust setae in distal row; propodus length 2.3 times width, with 5 robust setae on posterior margin. Pereopod 2 (Fig. 3B, C) ischium with 3 robust setae on posterior margin and 2 robust setae on posterodistal margin; merus with 7 short robust setae on anterodistal angle, posteromedial margin with 3 robust setae in proximal row and 2 robust setae in distal row; propodus with 4 robust setae on posterior margin. Pereopod 7 coxa (Fig. 2F) distally attenuated, curved posteriorly.

Pleonite 3 (Fig. 2F) not extending beyond pleonite 5.

Uropod (Figs 2D, 3D, E) not extending beyond pleotelson; peduncle with 3 robust setae; exopod and endopod with smooth lateral and distal margins; exopodal lateral margin convex with 10 robust setae along margin, setal fringe medium to continuous in length (69%), medial margin straight, distomedial corner rounded, distal margin convex with 5 robust setae, distolateral corner slightly produced, acute; endopodal lateral margin convex, distally sinuate, with 4 robust setae; medial margin straight; distomedial corner rounded; distal margin straight with 11 robust setae; distolateral corner produced, acute.

Pleotelson (Fig. 2D) broader than long, 1.6 times as wide as length, posterior margin medially concave, smooth (minute pores), conspicuous longitudinal carina on dorsal surface, with 11 distal and 2 lateral straight acute prominent spines along distal margin, without setae between spines, central distal spine simple.

#### Female.

Similar to male.

#### Variation.

Robust setae count on female as follows: exopodal lateral margin with 7–10 robust setae, distal margin with 4 or 5, endopodal lateral margin with 3–5 and distal margin with 8–10; pleotelson with 9 distal and 2 lateral straight acute prominent spines along distal margin.

#### Etymology.

The epithet is the Indonesian word “raksasa” for giant, alluding to its enormous size and the significance of the find. The name is used as a noun in apposition.

#### Remarks.

*Bathynomus raksasa* sp. nov. can be readily identified by its large size (330 mm on average), narrowly rounded clypeus apex, produced and acute distolateral corners of uropodal rami, wider rather than long pleotelson with medially concave posterior margin and the presence of 11–13 pleotelson spines. *Bathynomus raksasa* sp. nov. is the sixth “supergiant” species from the Indo-West Pacific and is one of the largest known members of the genus.

**Figure 4.**
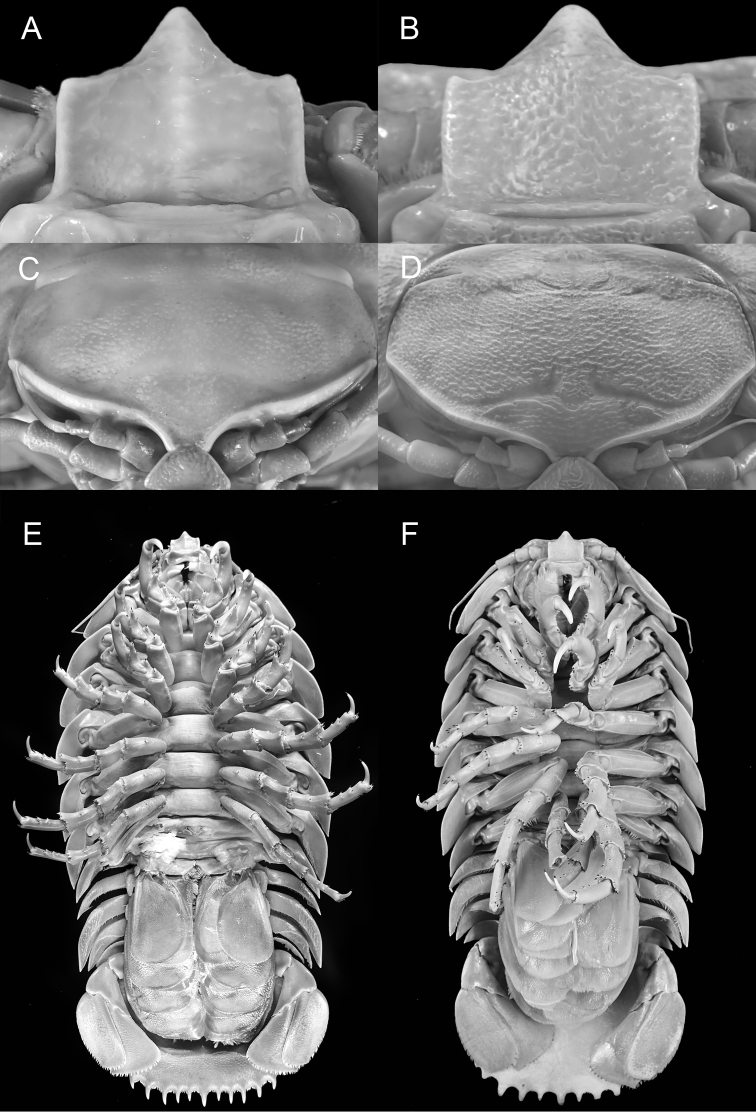
**A, C, E***Bathynomus raksasa* sp. nov. holotype male (363 mm) (MZB Cru.Iso 097), Indonesia **B, D, F***B. giganteus* male (354 mm) (ZRC 2014.0837), Caribbean **A, B** clypeus ventral surface **C, D** anterior of head **E, F** body, ventral view.

**Figure 5.**
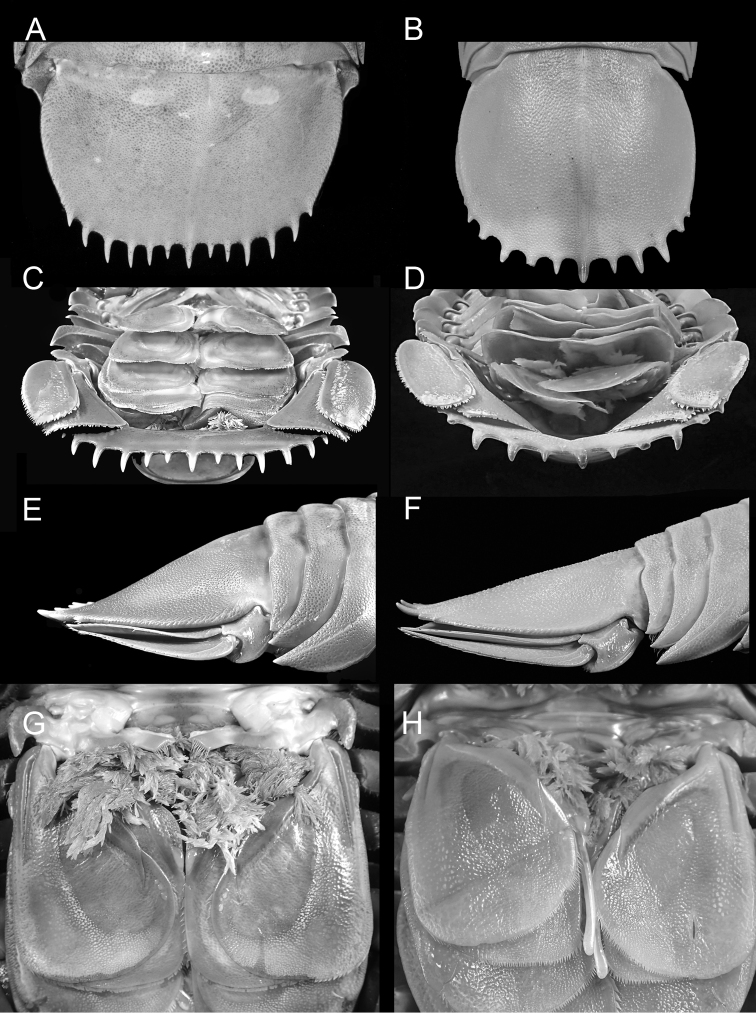
**A, C, E, G***Bathynomus raksasa* sp. nov. holotype male (363 mm) (MZB Cru.Iso 097), Indonesia **B, D, F, H***B. giganteus* male (354 mm) (ZRC 2014.0837), Caribbean **A, B** pleotelson dorsal view **C, D** pleotelson posterior view **E, F** pleotelson lateral view **G, H** pleopod 2.

In general appearance, *B. raksasa* sp. nov. is most similar to *B. giganteus* and *B. lowryi*. All three are large, averaging 300 mm in length, possess a prominent longitudinal carina on the dorsal surface of the pleotelson and have acute spines on the distal margin of the pleotelson. The new species is closest to *B. giganteus*, sharing the relatively medium length of antenna 2 (reaching to between the posterior of pereonite 2 and anterior of pereonite 3), lateral and posterior shape of the uropodal exopod and endopod, and the pleotelson spine count. *Bathynomus raksasa* sp. nov., however, differs markedly from *B. giganteus* by its more conspicuous longitudinal carina on the clypeus ventral surface (Fig. 4A) (vs. less conspicuous in *B. giganteus*; Fig. 4B), absence of a transverse carina on the anterior of the head (Fig. 4C) (vs. carina present in *B. giganteus*; Fig. 4D), the relatively shorter uropodal endopod (0.12 total body length, Fig. 4E) (vs. relatively longer, 0.15 body length in *B. giganteus*; Fig. 4F), the body surface, including that of the pleotelson, being covered with small low granules and smooth to the touch (Fig. 5A) (vs. granules more prominent and the surfaces distinctly rough in *B. giganteus*; Fig. 5B), the almost flat posterior ventral surface of the pleotelson (Fig. 5C) (vs. surface distinctly concave in *B. giganteus*; Fig. 5D), the straight spines of pleotelson (Fig. 5E) (vs. gently curved upwards in *B. giganteus*; Fig. 5F), the pleotelson is broader than long (Fig. 5A) (vs. as long as broad in *B. giganteus*, Fig. 5B), and the posterior margin of the pleotelson is broad and medially concave (Fig. 5A) (vs. broadly rounded in *B. giganteus*, Fig. 5B). *Bathynomus raksasa* sp. nov. can easily be distinguished from *B. lowryi* in possessing a relatively longer antenna 2 which reaches to the ends of pereonite 2 (vs. shorter antenna 2 which reaches only to the anterior part of pereonite 2 in *B. lowryi*), the narrowly rounded clypeus apex (vs. apex truncate in *B. lowryi*), straight pleotelson spines (vs. spines upwardly curved in *B. lowryi*) and the larger number (13) of robust setae on the pleotelson (vs. 9 in *B. lowryi*) (Bruce and Bussarawit 2004: figs 1, 6).

*Bathynomus raksasa* sp. nov. shares the same general uropodal exopod and endopod shape as *B. crosnieri*, *B. kensleyi* and *B. richeri* but can easily be distinguished from them in its possession of a conspicuous longitudinal carina on the dorsal surface of the pleotelson (Fig. 5A). Although the number of spines on the margin of the pleotelson (at least 11) is similar to those of *B. crosnieri* and *B. richeri*, the presence of the longitudinal ridge on the pleotelson easily separates *B. raksasa* sp. nov. from these species. *Bathynomus raksasa* sp. nov. also has the same number of spines on the margin of the pleotelson but can easily be distinguished from *B. keablei* in having the distolateral corners of the uropodal exopod and endopod distinctly produced (Fig. 3D, E) (vs. rounded and not produced in *B. keablei*; see Lowry and Dempsey 2006: fig. 17).

The appendix masculina is absent on the holotype male of *B. raksasa* sp. nov. (Fig. 5G) but this is almost certainly not a species-character. It is known to be sometimes absent in *B. doederleini* from Taiwan (present study); with five out of seven males below the size of 130 mm lacking it. The largest males of *B. doederleini* (136–145 mm) possess appendix masculina. The absence or presence of appendix masculina has been previously used by Soong and Mok (1994) to determine the maturity of males of *Bathynomus doederleini*; “mature males” were males with appendix masculina and “maturing males” were those without appendix masculina and testes. Barradas-Ortiz et al. (2003) reported that some adult males of *B. giganteus* from Brazil (mostly smaller specimens below 290 mm) lacked appendix masculina, especially in summer. They suggested that these smaller males might have been less reproductively active in summer and/or the appendix masculina may be a non-permanent organ which is lost or regrown when the animals moult (Barradas-Ortiz et al. 2003). Barradas-Ortiz et al. (2003) also noted that larger male specimens of *B. giganteus* tend to keep the organ for longer periods than smaller ones, although even large individuals (310 mm) sometimes do not possess the structure. We cannot be certain that either of the patterns above apply to *B. raksasa* sp. nov. as only one male was collected. The appendix masculina (Fig. 5H) is present on the large male American specimen of *B. giganteus* (ZRC 2014.0837) examined here.

**Figure 6.**
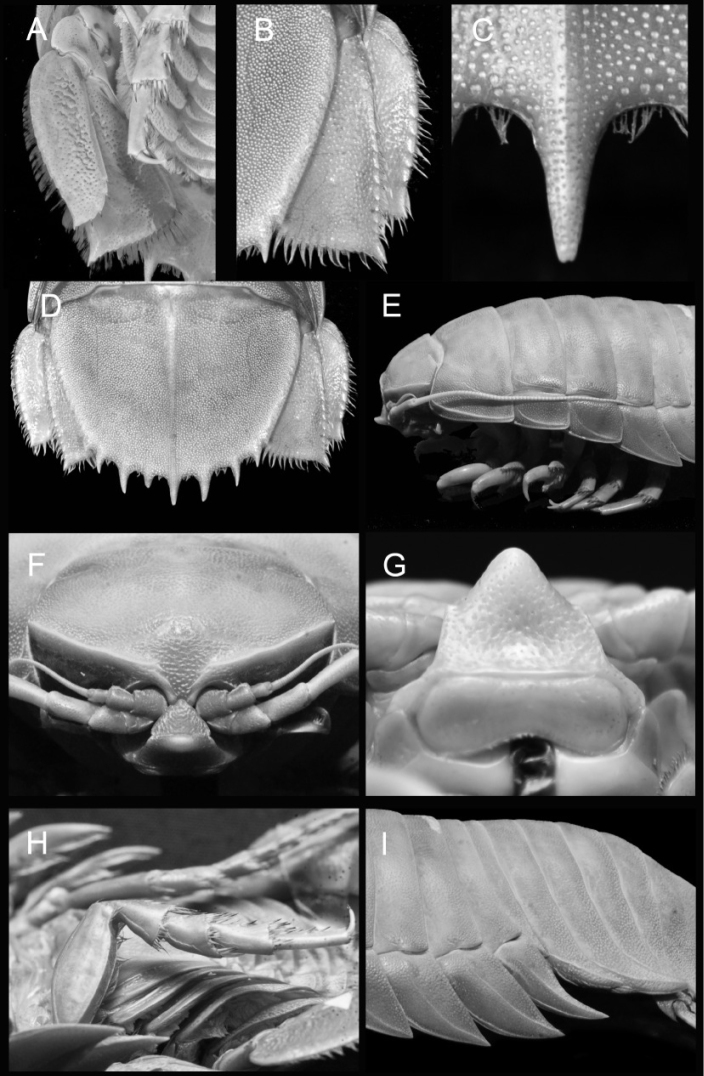
*Bathynomus* sp. (107 mm) (MZB Cru.Iso 098), Indonesia **A** uropod, ventral view **B** uropod, dorsal view **C** pleotelson central spine **D** pleotelson **E** length of antenna 2 **F** cephalon, anterior view **G** clypeal region **H** pereopod 7, ventral view **I** pereopod 7 coxa.

The SJADES cruise also obtained four juvenile and subadult specimens from southern Java (here identified as *Bathynomus* sp.) (Fig. 6) which we are unable to identify to the species level, especially as the diagnostic characters may not be developed. They are clearly not *Bathynomus raksasa* sp. nov. with a different pleotelson spination, shapes of pleotelson and uropodal rami. The largest specimen in the lot (107 mm) has an almost fully-developed pereopod 7 which indicates that the adult would not be too much larger in size. This, along with the presence of setae between the pleotelson spines, suggest that this species belongs to the “giant” group. The number of spines on the posterior margin of the pleotelson ranges between 5+2, 7+2 and 9+2. Soong and Mok (1994) used the development of pereopod 7 as one of the characters to classify the development stages of *Bathynomus doederleini*. According to Soong and Mok (1994), individuals with “small, white” pereopod 7 and lacking either oostegites or penes and/or appendix masculina were categorised as “subadult I” which equals to stage 2 of five development stages they proposed. However, we will not apply this approach to *Bathynomus* sp. because of the limited specimen number.

*Bathynomus* sp. superficially resembles the poorly known *Bathynomus affinis* Richardson, 1910, described from the Philippines from one specimen. There is, however, a problem with what has been identified as “*Bathynomus affinis*” by Lowry and Dempsey (2006: 169, figs 2, 3), who listed among their material, the type from the Philippines as well as two females from the Arafura Sea, providing figures of the latter. Bruce (1986: fig. 87A–E) had earlier figured the uropods, and pereopods 1 and 3 of the type specimen (sex not specified). The problem is that the distolateral corners of uropodal rami of the holotype from the Philippines is distinctly acute and curved (Bruce 1986: fig. 87A–C) whereas that of Lowry and Dempsey (2006: fig. 3D, E) from the Arafura Sea is distinctly wider and not produced. Significantly, Richardson’s (1910: fig. 1) figures of the uropods are the same as those by Bruce (1986). The material from Arafura Sea are thus unlikely to be *B. affinis* s. str.

Our material of *Bathynomus* sp. from Java resembles the “*B. affinis*” of Lowry and Dempsey (2006) in possessing the same relative length of antenna 2 (reaching between pereonites 3 and 4), straight clypeus distal margins, the setal fringe on the uropodal exopod is long and continuous (± 90%), and similar pleotelson spine count (5+2, 7+2 and 9+2). The marked difference in the form of the uropodal endopod distolateral corner, however, indicates they are not conspecific. In addition, the uropod of *Bathynomus* sp. reaches to the end of the pleotelson (Fig. 6D) (vs. slightly extended beyond the pleotelson; Lowry and Dempsey 2006: fig. 2F) and the pleotelson central spine is weakly bifid (Fig. 6C) (vs. simple; Lowry and Dempsey 2006: fig. 2F). The uropods of our material from Java agree very well with the figures by Richardson (1910) and Bruce (1986), but until a complete redescription of the holotype of *B. affinis* is done and more character states are known, we are not certain if they are actually conspecific.

*Bathynomus* sp. differs from *B. pelor* Bruce, 1986 (from northwestern Australia) in having a longer antenna 2 that reaches to the middle of pereonite 4 (Fig. 6E) (vs. middle of pereonite 2; Bruce 1986: fig. 91A), weakly bifid pleotelson central spine (Fig. 6C) (vs. strongly bifid; Bruce 1986: fig. 91B), and the conspicuous longitudinal carina on the pleotelson (Fig. 6D) (vs. inconspicuous; Bruce 1986: fig. 91C). Both species share similar shape of uropodal rami with more acute and curved distolateral corner on the endopod of *Bathynomus* sp. (Fig. 6A, B) (vs. less acute and curved; Bruce 1986: fig. 91D). It differs from *B. immanis* Bruce, 1986, in the slightly concave lateral of uropodal exopod (Fig. 6A, B) (vs. strongly concave; Bruce 1986: fig. 90C, D), greater length of fringing setae (± 80%) on the lateral uropod exopod (Fig. 6A, B) (vs. 66%; Bruce 1986: fig. 90C, D) and the weakly bifid pleotelson central spine (Fig. 6C) (vs. simple; Bruce 1986: fig. 89 D). The two species together with *B. doederleini* share similar uropodal endopod shapes (Fig. 6A, B).

*Bathynomus* sp. shares with *B. kapala* Griffin, 1975 (from Australia) a similar bifid central pleotelson spine but can easily be distinguished by its relatively longer antenna 2 (Fig. 6E) (middle of pereonite 4 vs. within pereonite 3; Lowry and Dempsey 2006: fig. 14 C), the straight head ridge (Fig. 6F) (vs. curved; Lowry and Dempsey 2006: fig. 14 D), a narrowly rounded clypeus apex (6G) (vs. broadly rounded; Lowry and Dempsey 2006: fig. 14 E), with only one row of fringing setae on the anterior margin of the basis of pereopod 7 (Fig. 6H) (vs. with two rows; Lowry and Dempsey 2006: fig. 23 F) and the uropodal endopod distolateral margin is subacute and only slightly produced (Fig. 6A, B) (vs. not produced; Lowry and Dempsey 2006: fig. 15 D, E).

Compared to *B. doederleini*, *Bathynomus* sp. has pereopod 7 coxa more slender (Fig. 6I) (vs. relatively broader; Lowry and Dempsey 2006: fig. 10B), there is one row of fringing setae on the anterior margin of the basis of pereopod 7 (Fig. 6H) (vs. with two rows; Lowry and Dempsey 2006: fig. 23D), and the lengths of the pleotelson spines are similarly sized (Fig. 6D) (vs. uneven; Lowry and Dempsey 2006: fig. 10F).

#### Distribution.

Sunda Strait and Indian Ocean, South Java, Indonesia; at depths of 957–1259 m.

## Supplementary Material

XML Treatment for Bathynomus

XML Treatment for Bathynomus raksasa
